# Supplementary material for: Comparative Study of the Co-Occurring Alternaria and Colletotrichum Species in the Production of Citrus Leaf Spot
Source: J Fungi (Basel). 2023 Nov 8;9(11):1089. doi: 10.3390/jof9111089 (PMC10672444; doi:10.3390/jof9111089)
Supplement: Supplementary file 1 [file jof-09-01089-s001.zip › jof-2656705-supplementary.pdf]

Table S1. The information of transcriptional sequencing of Gonggan leaves.

Sample name	Inoculant	Time point (h)	Total reads	GC content	Q30 ratio	Mapped reads	Uniq mapped reads	Multiple mapped reads	Reads mapped to '+'	Reads mapped to '-'
A1	PDA disc	48	51,543,282	44.10%	95.03%	47,258,195 (91.69%)	45,921,877 (89.09%)	1,336,318 (2.59%)	24,452,646 (47.44%)	24,449,633 (47.44%)
A2	PDA disc	48	51,026,572	44.05%	95.12%	47,948,208 (93.97%)	46,601,424 (91.33%)	1,346,784 (2.64%)	24,821,291 (48.64%)	24,807,686 (48.62%)
A3	PDA disc	48	48,591,390	44.17%	95.28%	45,451,685 (93.54%)	44,160,862 (90.88%)	1,290,823 (2.66%)	23,535,957 (48.44%)	23,533,756 (48.43%)
A4	PDA disc	48	50,739,488	44.10%	95.42%	47,661,474 (93.93%)	46,260,448 (91.17%)	1,401,026 (2.76%)	24,701,873 (48.68%)	24,692,819 (48.67%)
A5	Colletotrichum F12C	48	50,236,564	44.25%	95.35%	47,218,368 (93.99%)	45,653,376 (90.88%)	1,564,992 (3.12%)	24,580,868 (48.93%)	24,564,353 (48.90%)
A6	Colletotrichum F12C	48	47,741,886	44.40%	95.39%	44,969,182 (94.19%)	43,262,394 (90.62%)	1,706,788 (3.58%)	23,519,143 (49.26%)	23,524,327 (49.27%)
A7	Colletotrichum F12C	48	47,706,772	44.31%	96.13%	45,122,040 (94.58%)	43,600,790 (91.39%)	1,521,250 (3.19%)	23,494,459 (49.25%)	23,506,823 (49.27%)
A8	Colletotrichum F12C	48	47,556,438	44.41%	95.59%	44,487,428 (93.55%)	42,983,400 (90.38%)	1,504,028 (3.16%)	23,189,308 (48.76%)	23,166,620 (48.71%)
A9	Alternaria F12A	48	50,983,214	44.27%	95.57%	47,747,897 (93.65%)	46,059,963 (90.34%)	1,687,934 (3.31%)	24,948,760 (48.94%)	24,900,878 (48.84%)
A10	Alternaria F12A	48	47,136,454	44.08%	95.85%	44,482,707 (94.37%)	43,054,835 (91.34%)	1,427,872 (3.03%)	23,158,288 (49.13%)	23,109,482 (49.03%)
A11	Alternaria F12A	48	47,940,984	43.92%	95.22%	44,649,014 (93.13%)	43,322,337 (90.37%)	1,326,677 (2.77%)	23,177,641 (48.35%)	23,134,627 (48.26%)
A12	Alternaria F12A	48	47,989,314	44.41%	95.76%	44,323,731 (92.36%)	42,854,440 (89.30%)	1,469,291 (3.06%)	23,082,526 (48.10%)	23,068,007 (48.07%)
B1	PDA disc	72	48,144,656	44.01%	95.44%	45,237,033 (93.96%)	43,949,642 (91.29%)	1,287,391 (2.67%)	23,404,761 (48.61%)	23,412,504 (48.63%)
B2	PDA disc	72	49,184,682	44.21%	95.43%	46,488,064 (94.52%)	45,212,150 (91.92%)	1,275,914 (2.59%)	24,045,637 (48.89%)	24,034,737 (48.87%)
B3	PDA disc	72	48,292,000	44.32%	95.61%	45,410,408 (94.03%)	43,827,829 (90.76%)	1,582,579 (3.28%)	23,674,137 (49.02%)	23,697,209 (49.07%)
B4	PDA disc	72	52,198,076	44.08%	94.56%	48,466,686 (92.85%)	47,009,502 (90.06%)	1,457,184 (2.79%)	25,127,902 (48.14%)	25,124,807 (48.13%)
B5	Colletotrichum F12C	72	50,480,064	44.17%	95.51%	47,200,364 (93.50%)	45,792,766 (90.71%)	1,407,598 (2.79%)	24,476,488 (48.49%)	24,450,922 (48.44%)
B6	Colletotrichum F12C	72	47,609,200	44.43%	95.76%	43,655,478 (91.70%)	42,307,645 (88.86%)	1,347,833 (2.83%)	22,653,657 (47.58%)	22,644,278 (47.56%)
B7	Colletotrichum F12C	72	49,021,102	44.26%	95.49%	46,120,564 (94.08%)	44,280,946 (90.33%)	1,839,618 (3.75%)	24,181,416 (49.33%)	24,203,810 (49.37%)
B8	Colletotrichum F12C	72	48,493,636	44.09%	95.51%	45,466,801 (93.76%)	43,825,392 (90.37%)	1,641,409 (3.38%)	23,755,822 (48.99%)	23,767,508 (49.01%)
B9	Alternaria F12A	72	48,861,856	44.18%	95.12%	45,981,482 (94.11%)	44,361,521 (90.79%)	1,619,961 (3.32%)	23,988,495 (49.09%)	23,968,695 (49.05%)
B10	Alternaria F12A	72	50,702,244	43.81%	95.78%	47,609,534 (93.90%)	46,158,885 (91.04%)	1,450,649 (2.86%)	24,710,592 (48.74%)	24,669,302 (48.66%)
B11	Alternaria F12A	72	55,608,346	44.17%	95.67%	52,262,814 (93.98%)	50,544,061 (90.89%)	1,718,753 (3.09%)	27,197,794 (48.91%)	27,173,579 (48.87%)

B12	Alternaria F12A	72	56,462,636	43.91%	95.62%	51,029,819 (90.38%)	49,443,897 (87.57%)	1,585,922 (2.81%)	26,511,823 (46.95%)	26,469,627 (46.88%)

'+', the DNA sense chain; '-', the DNA antisense chain.
